# FROM PROGRAM PARTICIPANT TO AGING ADVOCATE: THEORIZING OLDER ADULTS' ROLES IN AGE-FRIENDLY COMMUNITY INITIATIVES

**DOI:** 10.1093/geroni/igz038.097

**Published:** 2019-11-08

**Authors:** Emily Greenfield, Laurent Reyes

**Affiliations:** 1 Rutgers University, New Brunswick, New Jersey, United States; 2 Rutgers, The State University Of New Jersey, New Brunswick, New Jersey, United States

## Abstract

Major professional organizations in health and aging have identified older adults’ involvement as a defining feature of aging-friendly community initiatives (AFCIs), yet there is very little research on this aspect of the initiatives. Our study utilizes five waves of data from in-depth interviews with leaders of nine AFCIs across northern New Jersey. The study was conducted as part of a multi-year, community-partnered project on the development of these philanthropically supported initiatives. Multi-phase coding yielded four types of roles for older adults: (a) program participants, (b) informants and consultants, (c) volunteers assisting with programs, events, and administrative tasks, and (d) aging-friendly champions and advocates. Across role types, AFCI leaders reflected on the challenges of engaging particular subgroups of older adults, as well as how ambivalent age identities impeded involvement. We discuss implications for advancing research and evaluation on community-level interventions that seek to simultaneously serve and empower people as they age.

